# Designing high-performance layered thermoelectric materials through orbital engineering

**DOI:** 10.1038/ncomms10892

**Published:** 2016-03-07

**Authors:** Jiawei Zhang, Lirong Song, Georg K. H. Madsen, Karl F. F. Fischer, Wenqing Zhang, Xun Shi, Bo B. Iversen

**Affiliations:** 1 Center for Materials Crystallography, Department of Chemistry and iNANO, Aarhus University, Langelandsgade 140, Aarhus DK-8000, Denmark; 2 Computational Materials Discovery, ICAMS, Ruhr-Universität Bochum, Bochum 44801, Germany; 3 State Key Laboratory of High Performance Ceramics and Superfine Microstructure, Shanghai Institute of Ceramics, Chinese Academy of Sciences, 1295 Dingxi Road, Shanghai 200050, China; 4 Materials Genome Institute, Shanghai University, Shanghai 200444, China

## Abstract

Thermoelectric technology, which possesses potential application in recycling industrial waste heat as energy, calls for novel high-performance materials. The systematic exploration of novel thermoelectric materials with excellent electronic transport properties is severely hindered by limited insight into the underlying bonding orbitals of atomic structures. Here we propose a simple yet successful strategy to discover and design high-performance layered thermoelectric materials through minimizing the crystal field splitting energy of orbitals to realize high orbital degeneracy. The approach naturally leads to design maps for optimizing the thermoelectric power factor through forming solid solutions and biaxial strain. Using this approach, we predict a series of potential thermoelectric candidates from layered CaAl_2_Si_2_-type Zintl compounds. Several of them contain nontoxic, low-cost and earth-abundant elements. Moreover, the approach can be extended to several other non-cubic materials, thereby substantially accelerating the screening and design of new thermoelectric materials.

## Abstract (web summary)

Thermoelectric materials with enhanced performances need to be identified. Here, the authors use the crystal field splitting energy of orbitals as a descriptor to design thermoelectric materials by solid solution maps and strain engineering in layered CaAl_2_Si_2_-type Zintl compounds.

## Graphical abstract

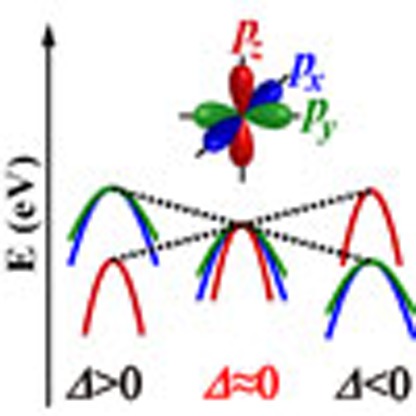

With the gradual depletion of fossil fuels and the growing pressure of the global warming, developing reliable and sustainable approaches for energy conversion is a key challenge for our society. Thermoelectric (TE) devices, which can directly and reversibly convert heat into electricity, have potential applications in waste-heat recovery, air conditioning and refrigeration^1^,^2^. However, the application of TE devices is critically limited by their low efficiencies, which are determined by the performance of the materials. Numerous efforts worldwide thus focus on optimizing current TE materials and developing novel TE materials with enhanced performance^3^,^4^,^5^,^6^,^7^,^8^,^9^. The performance of TE materials is governed by the dimensionless figure of merit, *zT=α*^2^*σT/κ*, where *α* is the Seebeck coefficient, *σ* is the electrical conductivity, *κ* is the thermal conductivity and *T* is the absolute temperature. Accordingly, enhanced *zT* value requires a combination of excellent electrical transport properties, quantified by the TE power factor PF*=α*^2^*σ*, and low thermal conductivity.

Several successful concepts, including nano-structuring^10^ and the inclusion of loosely bound rattlers^11^, have been developed to systematically reduce the lattice thermal conductivity. On the other hand, a systematic optimization of the electronic properties, which often exhibit a highly nontrivial dependence on atomic structure, still poses a big challenge. High-throughput computational screening allows the identification of compounds with high power factors via electronic transport calculations^12^,^13^. The physical insight from the approach is, however, mainly limited to the rationalization of the calculated properties. Furthermore, derived compounds, for example, a high-performing alloy of two low-performing compounds, can be overlooked. How alloying can optimize the PF by alignment of band edges has been demonstrated experimentally^5^,^14^,^15^ and theoretically^16^,^17^. However, despite the intuitive appeal of this approach, it has only been applied to a few cubic or pseudo-cubic alloys. This might be attributed to the limited insight into the underlying bonding orbitals at the band edges, which makes it difficult to extend the approach to new materials.

Here, taking layered CaAl_2_Si_2_-type Zintl compounds as an example, we combine first principles calculations and reported experimental data^18^,^19^,^20^,^21^,^22^,^23^,^24^,^25^,^26^,^27^,^28^,^29^,^30^,^31^,^32^,^33^ to demonstrate how the TE properties can be rationalized in terms of a simple crystal field scheme of orbitals splitting. Thereby, a powerful selection rule only based on band structure parameters is established as a simplified descriptor of electrical transport performance. Using the screening rule, we identify a few promising TE candidates with nontoxic, inexpensive and earth-abundant elements from CaAl_2_Si_2_-type Zintl compounds. It is shown how the selection rule naturally leads to strategies for rationally optimizing the TE PF through solid solution map and biaxial strain engineering. Finally, the strategy is reasonably extended to several other types of materials including chalcopyrite, metal dichalcogenide and lithium intercalated metal dichalcogenide. The orbital engineering approach presented here thus provides insightful guidance for the search and design of novel promising TE materials with good electronic transport properties.

## Results

### Orbital characteristics and electronic transport properties

**Figure 1:**
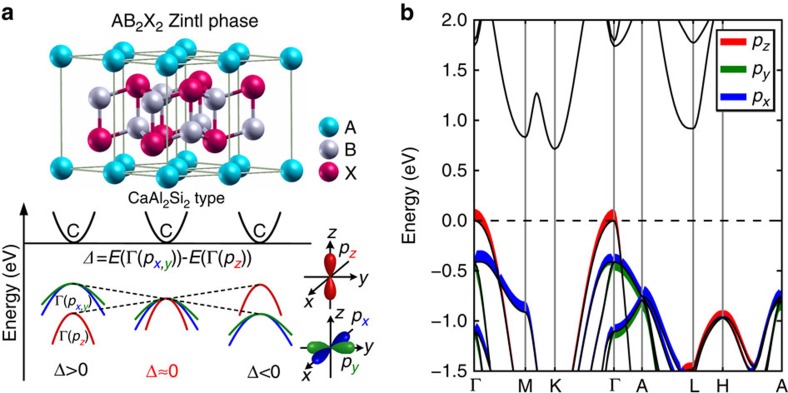
Orbital engineering to realize threefold degenerate *p* orbitals in layered CaAl_2_Si_2_-type Zintl compounds. (**a**) Crystal structure and electronic bands of CaAl_2_Si_2_-type Zintl compounds. Nondegenerate band Γ(*p*_*z*_) and doubly degenerate band Γ(*p*_*x,y*_) are mainly composed of *p*_*z*_ and *p*_*x,y*_ orbitals from anions, respectively. *Δ* is the crystal field splitting energy between *p*_*x,y*_ and *p*_*z*_ orbitals at the *Γ* point. (**b**) Orbital-projected band structure of representative CaAl_2_Si_2_-type Zintl compound Mg_3_Sb_2_ with negative* Δ* value. *p*_*x*_, *p*_*y*_ and *p*_*z*_ orbitals of Sb anions are projected on the band structure. Curve width indicates the relative weight of the component. Two representative compounds Mg_3_Sb_2_ (*Δ*<0) and SrZn_2_Sb_2_ (*Δ*>0, Supplementary Fig. 2) are used to demonstrate the *p* orbital characteristics of CaAl_2_Si_2_-type Zintl materials.

AB_2_X_2_ Zintl compounds crystallize in trigonal (space group: *P*
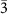
*m*1) CaAl_2_Si_2_-type structures, where A is an alkaline-earth or a divalent rare-earth element, B is a transition-metal or a main group element, X normally comes from group 15 and 14 (ref. 34). Generally, layered AB_2_X_2_ Zintl compounds can be described as trigonal monolayers of A^2+^ cations in the *a*-*b* plane separating [B_2_X_2_]^2−^ covalently bound slabs (Fig. 1a)^27^. These Zintl compounds are known by the virtue of low thermal conductivity^35^. Furthermore, the wide variety of compositions covered by this type of compounds provides considerable chemical tunability of the transport properties. However, their complicated chemical bonds, described in detail in ref. 34, make it a big challenge to systematically optimize the electronic transport properties.

The basic idea of the present paper is illustrated in Fig. 1a. The AB_2_X_2_ Zintl compounds are intrinsically p-doped^36^ and the electronic transport properties depend sensitively on the degeneracy of the valence band edges around the Brillouin zone centre at the Γ point. The valence band edge is dominated by the *p* orbital characteristics of the X anions (see Fig. 1b and Supplementary Figs 1 and 2 for details). Cubic symmetry would result in threefold degenerate *p* orbitals at Γ point because of the equivalency of the *x*, *y*, *z* directions in the Brillouin zone. However, owing to the effect of the crystal field splitting (see Supplementary Note 1 for details. The effect of spin orbit coupling is discussed in Supplementary Note 2.), the *p*_*z*_ orbital is separated from the *p*_*x*_ and *p*_*y*_ orbitals in layered materials. As a result, the valence bands at the Γ point split into a doubly degenerate band Γ(*p*_*x,y*_) and a nondegenerate band Γ(*p*_*z*_), mainly composed, respectively, of *p*_*x,y*_ and *p*_*z*_ orbitals from anions (Fig. 1). The energy difference between these two bands is defined as the crystal field splitting energy, namely, *Δ*=*E*(Γ(*p*_*x,y*_))−*E*(Γ(*p*_*z*_)). In general, Γ(*p*_*x,y*_) is a heavy hole band, whereas Γ(*p*_*z*_) is a light hole band. It is desirable for optimizing electrical transport performance that Γ(*p*_*x,y*_) band and Γ(*p*_*z*_) band are nearly degenerate^5^,^15^,^16^. Thus, the magnitude of *Δ* (|*Δ*|) can be used to evaluate the deviation from cubic-like threefold degeneracy of *p* orbitals at the band edges and thereby electrical transport properties. The deviation decreases as |*Δ*| gets smaller, and orbital degeneracy is effectively increased when *Δ* value is around zero. Therefore, orbital engineering is here understood as the reconstruction of cubic-like threefold degenerate *p* orbitals at the band edges in layered compounds through the manipulation of the relative energies of *p*_*x*_, *p*_*y*_ and *p*_*z*_ orbitals (see Fig. 1a for details).

**Figure 2:**
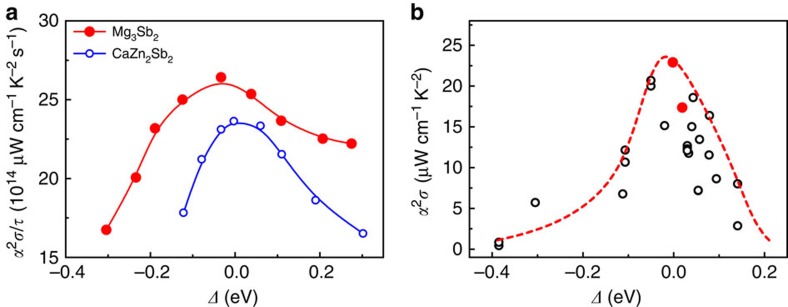
The dependence of thermoelectric power factors on crystal field splitting energy. (**a**) Calculated power factors *α*^2^*σ/τ* at 600 K of two representative CaAl_2_Si_2_-type Zintl compounds, Mg_3_Sb_2_ and CaZn_2_Sb_2_, with negative and positive *Δ*, respectively. The crystal field splitting energy *Δ* is varied by slightly tuning lattice parameters *a* and *c.* For each *Δ* value, the theoretical power factor *α*^2^*σ/τ* is calculated at a hole concentration *p*=10^20^ cm^−3^. A similar dependence of *α*^2^*σ/τ* on *Δ* is observed if the optimal carrier concentration is used (Supplementary Fig. 5). The solid lines represent fitted curves using a *B* spline. *τ* is the constant carrier relaxation time. (**b**) Experimental power factors^18^,^19^,^20^,^21^,^22^,^23^,^24^,^25^,^26^,^27^,^28^,^29^,^30^,^31^,^32^,^33^ *α*^2^*σ* at 600 K as a function of the crystal field splitting energy *Δ* in CaAl_2_Si_2_-type Zintl compounds. Solid solutions YbCd_1.6_Zn_0.4_Sb_2_ and EuZn_1.8_Cd_0.2_Sb_2_ with *zT* values^18^,^19^ above unity are marked in red. Experimental reported Zintl compounds^18^,^19^,^20^,^21^,^22^,^23^,^24^,^25^,^26^,^27^,^28^,^29^,^30^,^31^,^32^,^33^ studied here include YbCd_2_Sb_2_, YbZn_2_Sb_2_, EuZn_2_Sb_2_, EuCd_2_Sb_2_, CaZn_2_Sb_2_, SrZn_2_Sb_2_, CaMg_2_Bi_2_, YbMg_2_Bi_2_, Mg_3_Bi_2_, Mg_3_Sb_2_, Eu(Zn_1−*x*_Cd_*x*_)_2_Sb_2_ (*x*=0.1, 0.3 and 0.5), Yb(Zn_1−*x*_Cd_*x*_)_2_Sb_2_ (*x*=0.5 and 0.8) and Yb_1−*x*_Eu_*x*_Cd_2_Sb_2_ (*x*=0.25). The curve is guide to the eye, showing the best values corresponding to optimum carrier concentrations. The data points include varying carrier concentrations for the same compound reported in different references. Materials with carrier concentrations deviating from optimal values are below the curve.

**Figure 3:**
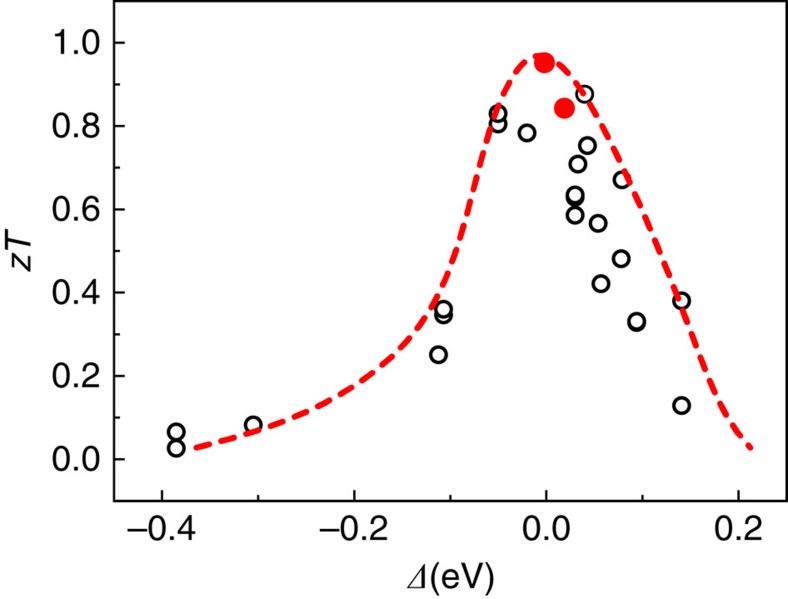
*zT* values at 600 K versus calculated *Δ* values in CaAl_2_Si_2_-type Zintl compounds. Experimental *zT* values are collected from refs 18, 19, 20, 21, 22, 23, 24, 25, 26, 27, 28, 29, 30, 31, 32, 33. YbCd_1.6_Zn_0.4_Sb_2_ and EuZn_1.8_Cd_0.2_Sb_2_ solid solutions with *zT* values^18^,^19^ above unity at high temperature are marked in red. The curve is guide to the eye, showing the maximum values corresponding to optimal carrier concentrations.

Figure 2a shows the calculated dependence of the PF on *Δ* for two representative CaAl_2_Si_2_-type Zintl compounds Mg_3_Sb_2_ and CaZn_2_Sb_2_ with, respectively, negative and positive *Δ* values (see also Supplementary Figs 3a, 4 and 5 for details). The theoretical power factors increase with the increasing *Δ* value and then decrease, showing peak values when *Δ* approaches zero, which is consistent with the above discussion. Figure 2b (see also Supplementary Fig. 3b for details) shows the experimental power factors of all reported TE Zintl compounds^18^,^19^,^20^,^21^,^22^,^23^,^24^,^25^,^26^,^27^,^28^,^29^,^30^,^31^,^32^,^33^ with the CaAl_2_Si_2_-type structure and their correlation with the calculated *Δ*. The result confirms that the peak power factors are obtained as *Δ≈*0, fully consistent with the trend of theoretical power factors. Moreover, the tendency of the experimental *zT* values at different temperatures shown in Fig. 3 and Supplementary Fig. 6 strongly resembles the dependence of the power factors on *Δ*. Considerably enhanced *zT* values are observed when *Δ* is around zero. In a few reported CaAl_2_Si_2_-type Zintl compounds with nearly zero *Δ* values, the optimum *zT* values^18^,^19^ of 1.0–1.2 are achieved at the temperature range of 600–700 K.

Moreover, the optimal electronic transport performance of TE materials is proportional to the weighted mobility, *μ*_0_(m*/*m*_e_)^3/2^, where *μ*_0_ is the intrinsic mobility, m* is the density-of-states effective mass and *m*_e_ is the mass of an electron^2^,^5^. Using the single parabolic band model and the assumption of acoustic phonon scattering mechanism, the values of *μ*_0_(m*/*m*_e_)^3/2^ and maximum *zT* at optimal carrier concentrations for many reported CaAl_2_Si_2_-type Zintl compounds^18^,^20^,^21^,^22^,^23^,^25^,^26^,^27^,^28^,^31^,^32^,^33^,^37^ are calculated from published Hall data (Supplementary Table 1). The correlation between *μ*_0_(m*/*m*_e_)^3/2^ (optimum *zT*) and *Δ* is demonstrated in Supplementary Fig. 7. As expected, *μ*_0_(m*/*m*_e_)^3/2^ (optimum *zT*) also undergoes a peak value when *Δ* is around zero.

### Screening rule for high electrical transport performance

Based on the established relationships between power factor, figure of merit (*zT*), orbital degeneracy and *Δ*, we are able to define a selection rule, that is, maintaining the crystal field splitting energy around zero (zero-*Δ* rule) to obtain good electrical transport performance and thereby high TE performance. Many CaAl_2_Si_2_-type Zintl compounds and corresponding *Δ* values are listed in Supplementary Table 2. Combining the selection criterion −0.06≤*Δ*≤0.06 and band gap *E*_g_<1.5 eV, we start the search for promising TE candidates from Zintl compounds. Besides those that have been confirmed by previous references^18^,^19^,^20^,^21^,^22^,^23^,^24^,^25^,^26^,^27^,^28^,^29^,^30^,^31^,^32^,^33^, compounds such as SrMg_2_Sb_2_, BaMg_2_Sb_2_, SrMg_2_Bi_2_ and BaMg_2_Bi_2_ are expected to show good electronic transport properties as well as TE performance. It is worthwhile to note that among these compounds potential TE candidates SrMg_2_Sb_2_ and BaMg_2_Sb_2_ contain earth-abundant, nontoxic and inexpensive elements.

The screening of a large number of CaAl_2_Si_2_-type Zintl compounds shows that only a few of the Zintl compounds possess nearly zero *Δ* values and suitable band gaps. It is therefore crucial to develop feasible and effective approaches to manipulate crystal field splitting energy for materials design and optimization. The splitting energy between the in-plane *p*_*x,y*_ and out-of-plane *p*_*z*_ orbitals at the valence band maximum is mainly determined by their hybridizations. Usually, compared with *p*_*x,y*_ orbitals, the energy level of *p*_*z*_ orbitals at the band edge will be more sensitive to their interlayer hybridization. Thus, tuning the interlayer distance could effectively manipulate the energy level of *p*_*z*_ orbitals and thereby crystal field splitting energy. Continuous change of the interlayer distance can be introduced by crystal deformation, which can be induced by both internally and externally applied forces. Internal forces involve chemical doping or forming solid solutions, whereas external forces include physical pressure, strain effect and so on. Here two powerful approaches, solid solution map and biaxial strain engineering, are developed to realize the tunability of *Δ*, which will be discussed below.

### Solid solution map for designing multinary TE compounds

**Figure 4:**
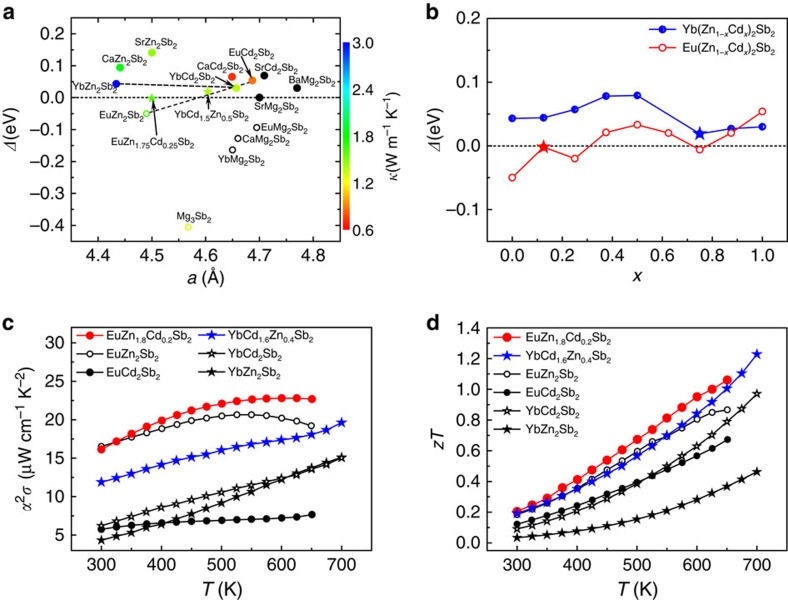
Solid solution map for designing high-performance multinary compounds with CaAl_2_Si_2_-type structure. (**a**) Calculated *Δ* as a function of the lattice constant *a* in CaAl_2_Si_2_-type Zintl compounds with *E*_g_<1.5 eV. The stars correspond to solid solutions YbCd_1.5_Zn_0.5_Sb_2_ and EuZn_1.75_Cd_0.25_Sb_2_ with nearly zero *Δ* values. Reported values^18^,^19^,^20^,^21^,^22^,^23^,^24^,^25^,^26^,^27^,^28^,^29^,^30^,^31^,^32^,^33^ of thermal conductivity at 500 K are shown in colour bar. (**b**) Calculated *Δ* versus the fraction *x* in two representative solid solutions Yb(Zn_1–*x*_Cd_*x*_)_2_Sb_2_ and Eu(Zn_1–*x*_Cd_*x*_)_2_Sb_2_. The stars represent solid solutions YbCd_1.5_Zn_0.5_Sb_2_ and EuZn_1.75_Cd_0.25_Sb_2_ with nearly zero *Δ* values. (**c**,**d**) Temperature dependence of power factors^18^,^19^ *α*^2^*σ* (**c**) and *zT* values^18^,^19^ (**d**) of YbZn_2_Sb_2_ (EuZn_2_Sb_2_), YbCd_2_Sb_2_ (EuCd_2_Sb_2_) and their solid solutions YbCd_1.6_Zn_0.4_Sb_2_ and EuZn_1.8_Cd_0.2_Sb_2_.

Figure 4a displays the *Δ* versus *a* compound map for CaAl_2_Si_2_-type Zintl compounds, where *a* is the lattice constant. The values of the thermal conductivity of reported Zintl compounds^18^,^19^,^20^,^21^,^22^,^23^,^24^,^25^,^26^,^27^,^28^,^29^,^30^,^31^,^32^,^33^ at 500 K are marked in the colour bar. Only a few compounds with band gaps smaller than 1.5 eV are illustrated out of the variety of Zintl compounds with CaAl_2_Si_2_-type structure. The compound maps as a function of, respectively, lattice constant *a* and band gap, with nearly all CaAl_2_Si_2_-type Zintl compounds are depicted in Supplementary Fig. 8a,b. In order to achieve an ideal *Δ*=0 value, one can select two or more compounds with positive (larger than 0) and negative (smaller than 0) *Δ* values and attempt to form a solid solution or mixture with *Δ*≈0 by slightly adjusting the molar ratio of the constituent compounds. This strategy constructs the overall picture of designing multinary CaAl_2_Si_2_-type Zintl compounds with ideal zero *Δ* values that yield good TE performance. Another possible optimizing approach is to select one compound with relatively low thermal conductivity and add it into one compound with nearly zero *Δ* forming a solid solution with the aim to block heat conduction without explicitly reducing the electronic transport through carefully tuning the molar ratio of the constituent compounds with poor heat conduction.

Through density functional theory calculation, *Δ* values as a function of the fraction *x* in two selected solid solutions Yb(Zn_1−*x*_Cd_*x*_)_2_Sb_2_ and Eu(Zn_1−*x*_Cd_*x*_)_2_Sb_2_ are simulated and shown in Fig. 4b. The results of Yb(Zn_1−*x*_Cd_*x*_)_2_Sb_2_ and Eu(Zn_1−*x*_Cd_*x*_)_2_Sb_2_ prove that it is indeed feasible to realize the ideal *Δ* value around zero in the selected solid solutions. The calculated band structure of an exemplified solid solution EuZn_1.75_Cd_0.25_Sb_2_ satisfying the nearly zero *Δ* value indicates that high orbital degeneracy at the valence band maxiamum is realized (Supplementary Fig. 9). Both Yb(Zn_1−*x*_Cd_*x*_)_2_Sb_2_ and Eu(Zn_1−*x*_Cd_*x*_)_2_Sb_2_ have been studied experimentally^18^,^19^. Figure 4c,d and Supplementary Fig. 10 show the temperature dependence of power factors, *zT* values, and thermal conductivities of two solid solutions YbCd_1.6_Zn_0.4_Sb_2_ and EuZn_1.8_Cd_0.2_Sb_2_ compared with the pure ternaries. The above two solid solutions with ideal *Δ* values around zero exhibit greatly enhanced power factors^18^,^19^ and *zT* values^18^,^19^ compared with non-alloyed compounds, consistent with the calculations. Moreover, the thermal conductivity of YbCd_1.6_Zn_0.4_Sb_2_ or EuZn_1.8_Cd_0.2_Sb_2_ is comparable to one of the constituent compounds. All the above results validate that the strategy based on solid solution maps is effective in the design of complex high-performance TE materials from a range of CaAl_2_Si_2_-type Zintl compounds. According to the well-established solid solution maps, there still exists many potential solid solutions that have not been reported yet, for example, Eu_1−*x*_Ca_*x*_Zn_2_Sb_2_, Yb_1−*x*_Ba_*x*_Mg_2_Sb_2_, Ca(Mg_1−*x*_Cd_*x*_)_2_Sb_2_, Yb(Mg_1−*x*_Zn_*x*_)_2_Sb_2_, Ca_1−*x*_Ba_*x*_Mg_2_Bi_2_ and so on. In particular, Ca(Zn_1−*x*_Mg_*x*_)_2_Sb_2_, Sr_1−*x*_Ba_*x*_Mg_2_Sb_2_ and Sr(Mg_1−*x*_Zn_*x*_)_2_Sb_2_ are promising TE candidates with earth-abundant, cheap and nontoxic chemical elements.

### Biaxial strain engineering to optimize *zT*

**Figure 5:**
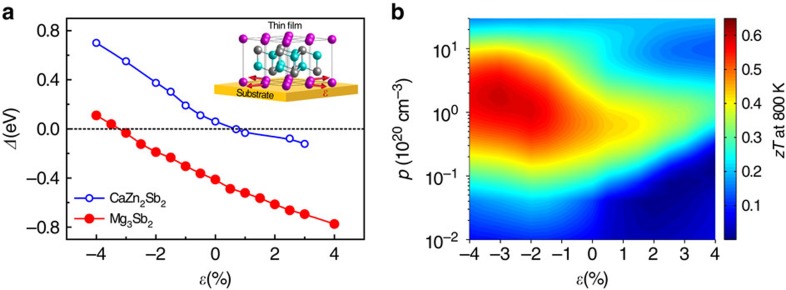
Biaxial strain engineering to optimize TE performance of CaAl_2_Si_2_-type Zintl compounds. (**a**) *Δ* versus biaxial strain *ɛ* in two representative CaAl_2_Si_2_-type Zintl compounds Mg_3_Sb_2_ and CaZn_2_Sb_2_. Here biaxial strain *ɛ* is defined as (*a*−*a*_0_)/*a*_0_ × 100%, where *a*_0_ and *a* are the in-plane lattice parameters with unstrained and strained states, respectively. (**b**) The contour map of calculated *zT* value of Mg_3_Sb_2_ at 800 K as a function of hole concentration *p* and biaxial strain ɛ.

In addition to the solid solution method, external forces like biaxial strain can also be used to manipulate the *Δ* value. The biaxial strain can be introduced here by the lattice mismatch between the substrate materials with selected cubic lattice and the thin film TE materials with the CaAl_2_Si_2_-type structure deposited on the substrate. The biaxial strain *ɛ* can be defined as (*a*-*a*_0_)/*a*_0_ × 100%, where *a*_0_ and *a* are the in-plane lattice parameters with unstrained and strained states, respectively. Figure 5a shows *Δ* as a function of *ɛ* in two representative CaAl_2_Si_2_-type Zintl compounds, Mg_3_Sb_2_ and CaZn_2_Sb_2_. As the figure depicts, a linear correlation between *Δ* and *ɛ* is observed. The value of *Δ* increases (decreases) linearly with the increasing magnitude of the compressive (tensile) strain. Thus, we can deduce a general optimization rule for high TE performance, that is, for Zintl compounds with positive *Δ* value tensile biaxial strain is more effective, whereas for Zintl compounds with negative *Δ* value compressive biaxial strain is preferred. According to the first-principles calculations, the calculated power factors can be continuously tuned by biaxial strain and show peak values at optimal biaxial strains corresponding to nearly zero *Δ* values (Supplementary Fig. 11). For negative-*Δ* Mg_3_Sb_2_, the optimal biaxial strain turns out to be compressive, whereas for positive-*Δ* CaZn_2_Sb_2_, optimal biaxial strain appears to be tensile, fully consistent with the above deduction. Using semiclassical Boltzmann transport theory and experimental data^30^ (see Methods for details), the dependence of *zT* at 800 K on carrier concentration and biaxial strain is estimated for Mg_3_Sb_2_ and plotted in Fig. 5b. The maximum *zT* value of Mg_3_Sb_2_ at 800 K at the optimal strain −3% shows around 50% enhancement compared with the value of the unstrained case. Thus, biaxial strain engineering is an effective approach for tuning and optimizing TE performance, showing potential application to thin-film materials with the CaAl_2_Si_2_-type structure.

### Extension of orbital engineering to several other compounds

**Figure 6:**
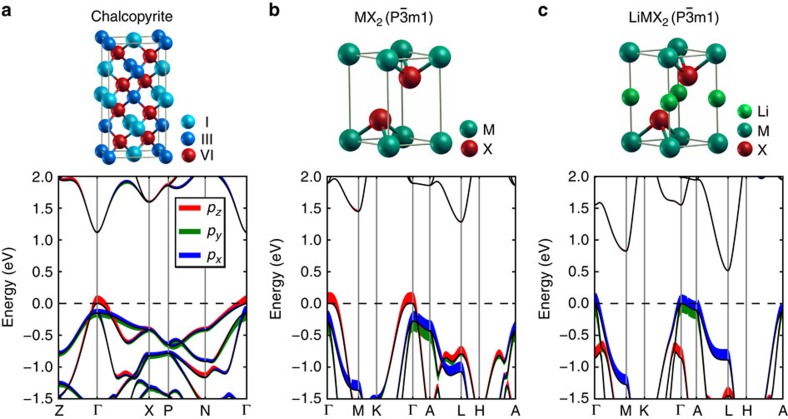
Extension of orbital engineering to several other non-cubic compounds. (**a**–**c**) Orbital-projected band structures and crystal structures of representative chalcopyrite compound AgGaTe_2_ (**a**), metal dichalcogenide ZrS_2_ (**b**) and lithium intercalated metal dichalcogenide LiZrSe_2_ (**c**). *p*_*x*_, *p*_*y*_ and *p*_*z*_ orbitals of anions are projected on the band structures. Curve width indicates the relative weight of the component.

The orbital engineering approach based on zero-*Δ* screening rule, solid solution map and biaxial strain engineering developed here can also be used for optimizing a vast number of non-cubic compounds with similar *p* orbitals characteristics at the band edges. Figure 6 and Supplementary Figure 12 show the orbital-projected band structures and density of states of AgGaTe_2_, ZrS_2_ and LiZrSe_2_, which are representatives of chalcopyrites, metal dichalcogenides MX_2_ (space group: *P*
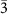
*m*1) and lithium intercalated metal dichalcogenides LiMX_2_ (space group: *P*
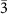
*m*1), respectively. All of these compounds possess crystal field split *p* orbital characteristics at the band edges, similar to the CaAl_2_Si_2_-type Zintl compounds. The results of biaxial strain engineering in chalcopyrites, metal dichalcogenides and lithium intercalated metal dichalcogenides from references (CuGaTe_2_ and TiS_2_)^38^,^39^ and our own data (ZrS_2_ and LiZrSe_2_; see Supplementary Fig. 13 for details) confirm orbital engineering. Detailed information including a solid solution map of metal dichalcogenides MX_2_ is provided in Supplementary Fig. 14 and Supplementary Table 3, with the aim to guide the optimization of *p*-type TE performance for metal dichalcogenides.

## Discussion

In summary, we have proposed a novel approach to realize high orbital degeneracy at the band edges that yields good electronic transport properties and thereby high TE performance via tuning the relative energies of orbitals in layered CaAl_2_Si_2_-type Zintl compounds. We establish a simple yet insightful screening criterion: maintaining crystal field splitting energy around zero. Using the calculated *Δ* versus lattice parameter maps, one can conveniently choose two or more compounds to form a solid solution with the desirable zero *Δ* value that leads to excellent electronic transport performance. Moreover, *Δ* and TE performance can be continuously tuned and optimized by biaxial strain engineering.

Based on the orbital engineering approach, we predict a series of potential TE candidates with CaAl_2_Si_2_-type structure. Some of them have been proved experimentally with enhanced *zT* values. Several predicted promising compounds contain earth-abundant, cheap and nontoxic elements. Finally, the orbital engineering strategy is rationally extended to other types of materials with the same orbital features at the band edges. Thus, we believe that orbital engineering strategy based on zero-*Δ* rule and augmented by solid solution map and biaxial strain engineering opens a new avenue for high-throughput computational screening of TE materials and thereby will accelerate the screening and design of new high-performance TE materials from a myriad of non-cubic compounds.

## Methods

### Computational methods

Density Functional Theory calculations were carried out using the projector-augmented wave method^40^ as implemented in the Vienna *ab initio* simulation package^41^. HSE06 functional^42^ was used to relax the internal coordinates in the unit cell, whereas the lattice parameters of CaAl_2_Si_2_-type Zintl compounds were fixed to the experimental values from the literature. Here HSE06 functional was used to get structural parameters close to the experimental values. The plane-wave energy cutoff was set at 400 eV, and a 6 × 6 × 4 Monkhorst-Pack *k* mesh was used for crystal structure optimization. A 12 × 12 × 8 Monkhorst-Pack *k* mesh and a 32 × 32 × 18 Monkhorst-Pack *k* mesh were used for electronic structure calculations and transport properties calculations, respectively. An energy convergence criterion of 10^−4^ eV and a Hellmann-Feynman force convergence criterion of 0.008 eV Å^−1^ were adopted. Electronic structure calculations were implemented by combining the mBJ potential and the well-established PBE+*U* method^43^,^44^, and *U*=4 eV (ref. 44) was applied on Zn 3*d* states and Cd 4*d* states. This method has already been successfully applied to a wide range of narrow gap semiconductors to get accurate band gaps comparable to the experimental values^43^,^44^. Electrical transport property calculations were carried out by combining the *ab initio* band structure calculations and the Boltzmann transport theory under the constant carrier scattering time approximation as implemented in the BoltzTraP^45^ code. Calculation details of electronic structure for solid solutions, chalcopyrite (AgGaTe_2_), metal dichalcogenides and lithium intercalated metal dichalcogenide (LiZrSe_2_) are given in Supplementary Notes 3–5. The calculation methods here are enough to study materials discussed in this paper (Supplementary Fig. 15), but might be insufficient to be used to study strongly correlated materials such as Kondo Insulators^46^ with strong hybridization of localized *f* electrons with electronic bands.

To study the effects of biaxial strain, a variety of in-plane *a* lattice parameters were analysed, and for each of them, the *c* parameter and the atomic positions were optimized. The carrier relaxation time *τ* was reasonably assumed to be independent of the strain in current work as the crystal structure for each step tuned by the biaxial strain is rather small. The figure of merit *zT* of Mg_3_Sb_2_ under biaxial strain effect (Fig. 5b) was calculated using the following formula:


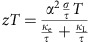


Where *α*, *σ*/*τ*, and *κ*_e_/*τ* can be directly obtained from BoltzTraP code. Thus, to obtain *zT*, we still need to know lattice thermal conductivity *κ*_L_ and constant scattering time *τ*. With the lattice thermal conductivity *κ*_L_(330 K)=1.341 W m^−1^ K^−1^ of Mg_3_Sb_2_ obtained from ref. 30, *κ*_L_(800 K)=0.553 W m^−1^ K^−1^ is estimated using the reciprocal relation of *κ*_L_ to *T*. Generally, under biaxial strain, lattice thermal conductivity is lower than that without strain because of the enhancement of phonon scattering by strain-induced defects^47^, which is beneficial to TE performance. To study the contribution to *zT* from electrical transport, lattice thermal conductivity here was assumed to be independent of biaxial strain. The carrier's scattering time *τ* could be derived from the relation *μ*=eτ/m*, where *μ* is the carrier mobility, m* is the effective mass of carrier and *e* is the elementary charge. Calculation details of constant carrier scattering time *τ* are provided in Supplementary Note 6 and Supplementary Table 4.

## Additional information

**How to cite this article:** Zhang, J. *et al.* Designing high-performance layered thermoelectric materials through orbital engineering. *Nat. Commun.* 7:10892 doi: 10.1038/ncomms10892 (2016).

## Supplementary Material

Supplementary Information Supplementary Figures 1-15, Supplementary Tables 1-4, Supplementary Notes 1-6 and Supplementary References
