# Supplementary material for: Designing high-performance layered thermoelectric materials through orbital engineering
Source: Nat Commun. 2016 Mar 7;7:10892. doi: 10.1038/ncomms10892 (PMC4786678; doi:10.1038/ncomms10892)
Supplement: Supplementary Information — Supplementary Figures 1-15, Supplementary Tables 1-4, Supplementary Notes 1-6 and Supplementary References [file ncomms10892-s1.pdf]

Supplementary Figures

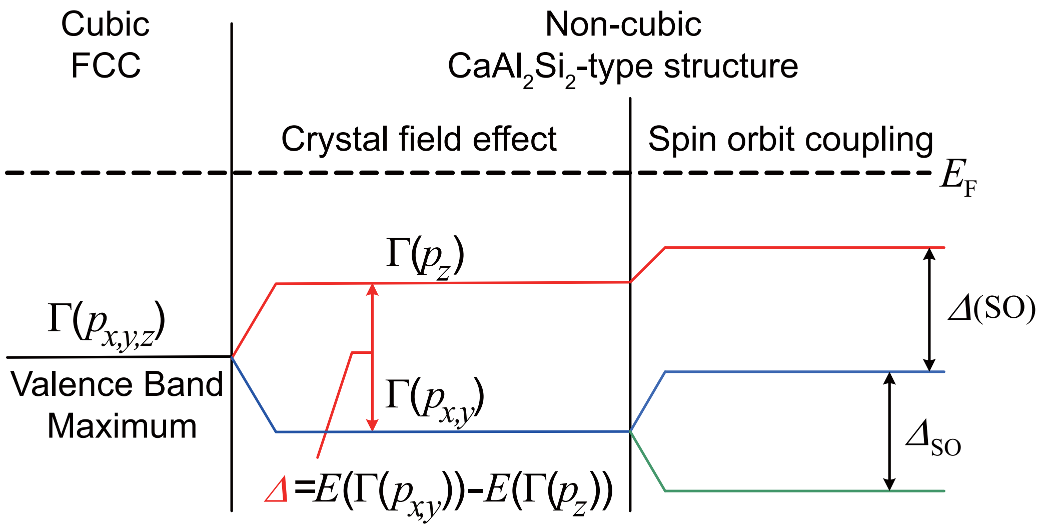

Supplementary Figure 1. Schematic diagram of the evolution from $p_{x,y,z}$ orbitals into valence bands at Γ point in $CaAl_2Si_2$-type Zintl compounds. The energy difference between top electronic state split from $p_{x,y}$ band and the electronic state from p_z band is defined as Δ(SO). The spin orbit splitting energy of $p_{x,y}$ band is labeled as Δ_{SO}.

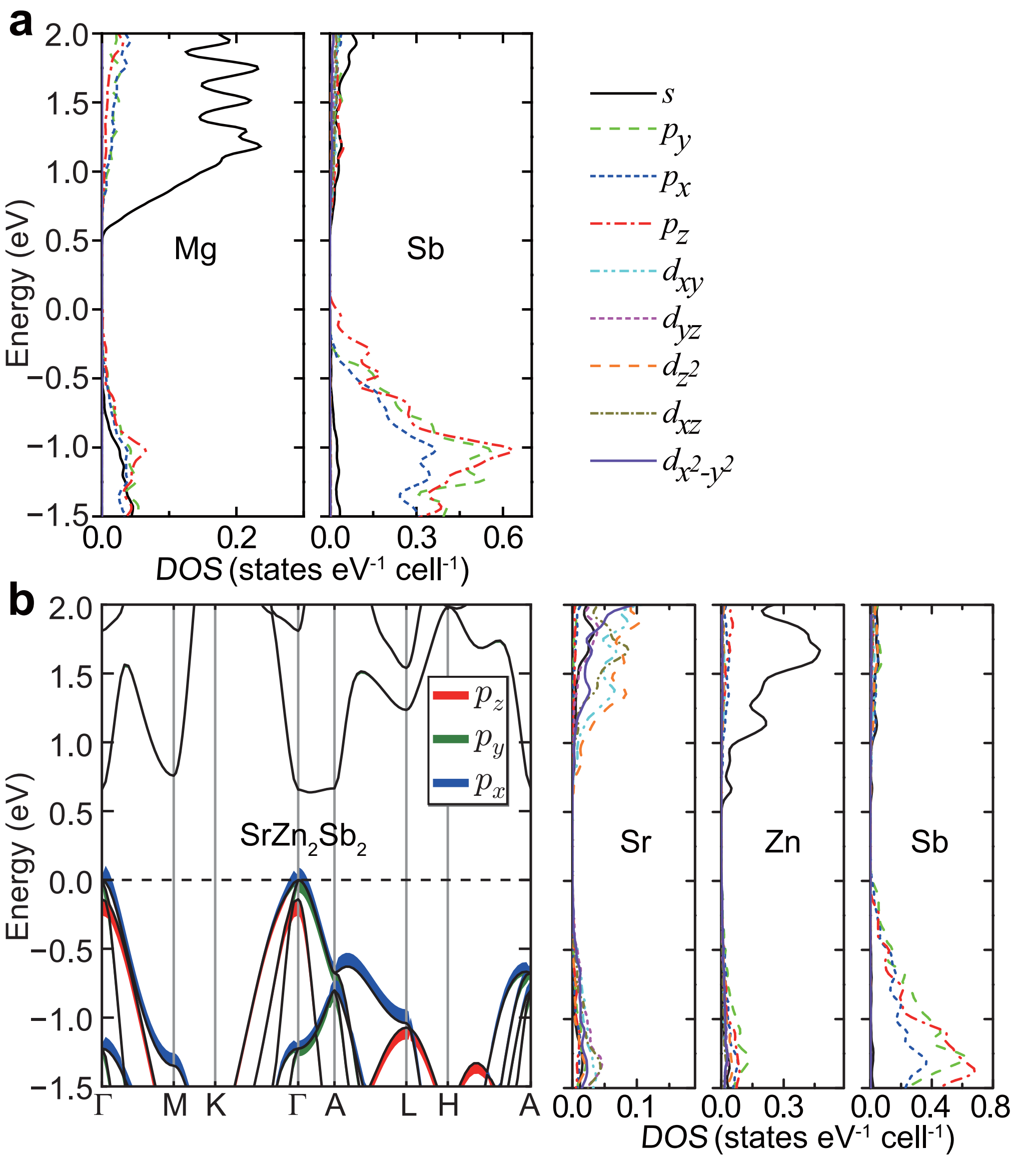

Supplementary Figure 2. (a) Orbital-projected density of states of Mg_3Sb_2. (b) Calculated orbital-projected band structures and density of states of representative $CaAl_2Si_2$-type Zintl compound $SrZn_2Sb_2$ with positive crystal field splitting energy *Δ*. p_x, p_y, and p_z orbitals of Sb anions are projected on the band structure. Curve width indicates the relative weight of the component.

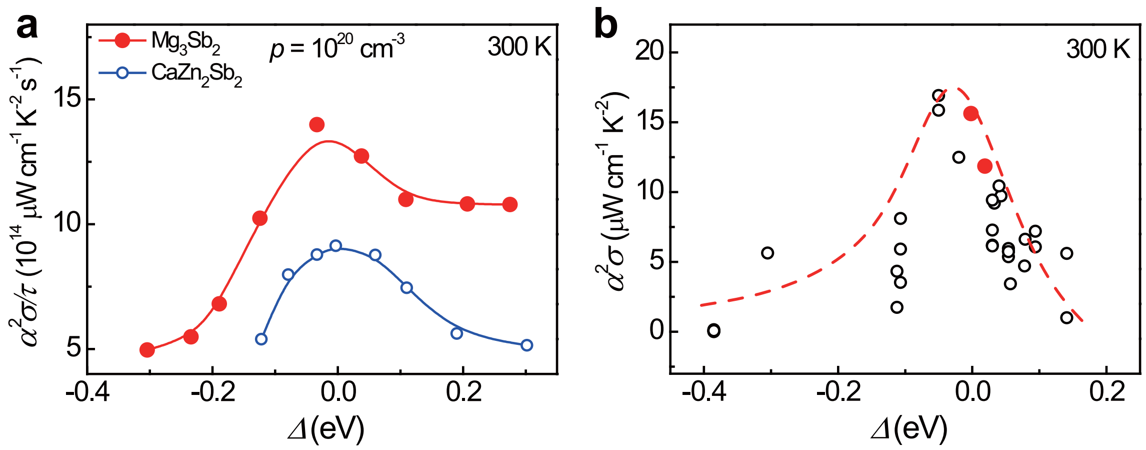

Supplementary Figure 3. (a) Calculated $\alpha^2\sigma/\tau$ ($p = 1\times10^{20}\text{cm}^{-3}$) and b) experimental power factors[1-16] ($\alpha^2\sigma$) at 300K as a function of the crystal field splitting energy Δ in $CaAl_2Si_2$-type Zintl compounds. The solid lines in Supplementary Fig. 3a represent fitted curves using a *B* spline. The curve in Supplementary Fig. 3b is guide to the eye, showing the maximum values corresponding to optimal carrier concentrations. Solid solutions $YbCd_{1.6}Zn_{0.4}Sb_2$ and $EuZn_{1.8}Cd_{0.2}Sb_2$ with *zT* values[2,3] above unity are marked in red.

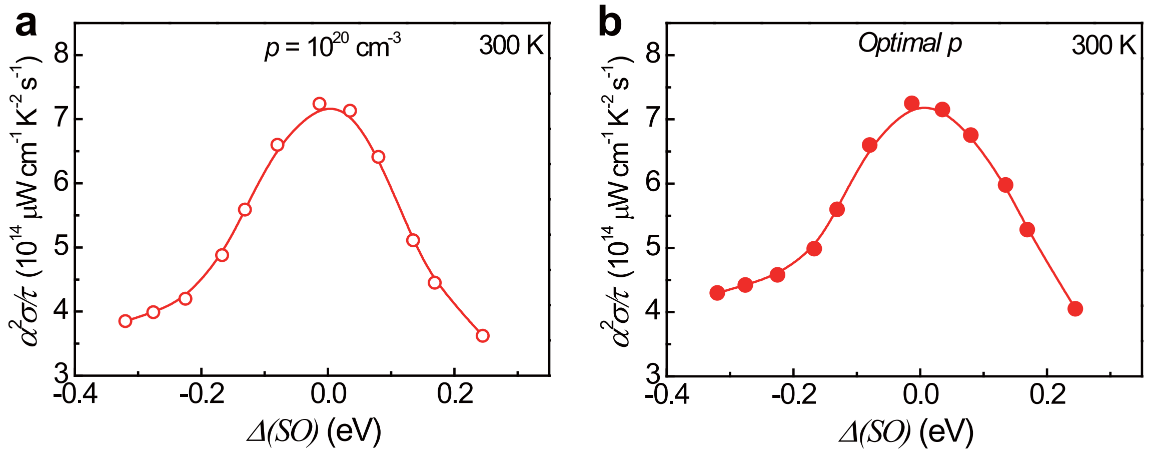

Supplementary Figure 4. Calculated room temperature theoretical $\alpha^2\sigma/\tau$ including spin orbit coupling effect at a given carrier concentration p = 1×10^{20} cm^{-3} (a) and optimal p (b) (10^{18}~10^{21} cm^{-3}) as a function of Δ(SO) in representative $CaAl_2Si_2$-type Zintl compound Mg_3Sb_2. The red lines represent fitted curves using a *B* spline.

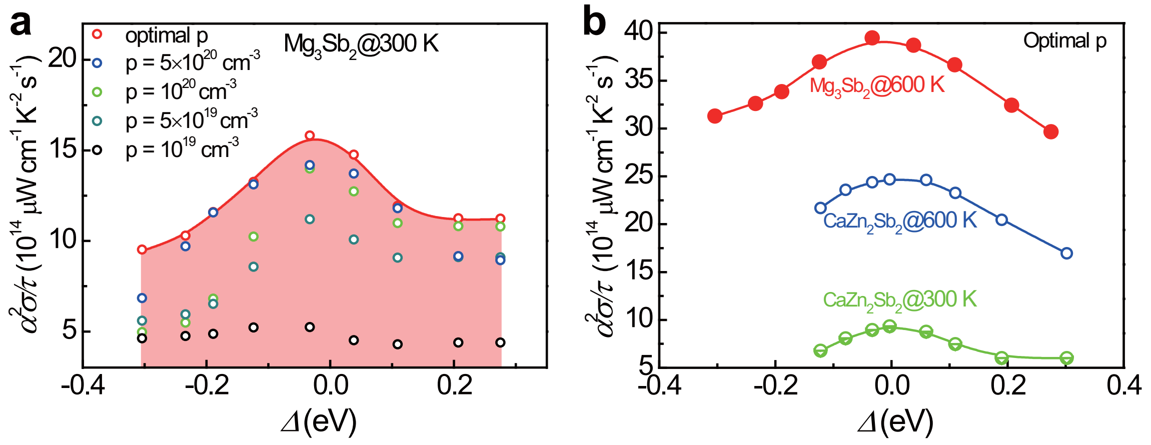

Supplementary Figure 5. (a) Calculated $\alpha^2\sigma/\tau$ of Mg_3Sb_2 at 300 K versus Δ at varying carrier concentrations (10^{19}~5×10^{20} cm^{-3}). The red curve shows the maximum values corresponding to optimal carrier concentrations. Data points for other unoptimized carrier concentrations fill up the whole pink area right below the best curve with optimal carrier concentration. (b) Calculated optimal $\alpha^2\sigma/\tau$ at 300 K and 600 K as a function of Δ in representative $CaAl_2Si_2$-type Zintl compounds Mg_3Sb_2 and $CaZn_2Sb_2$. The solid lines represent fitted curves using a *B* spline.

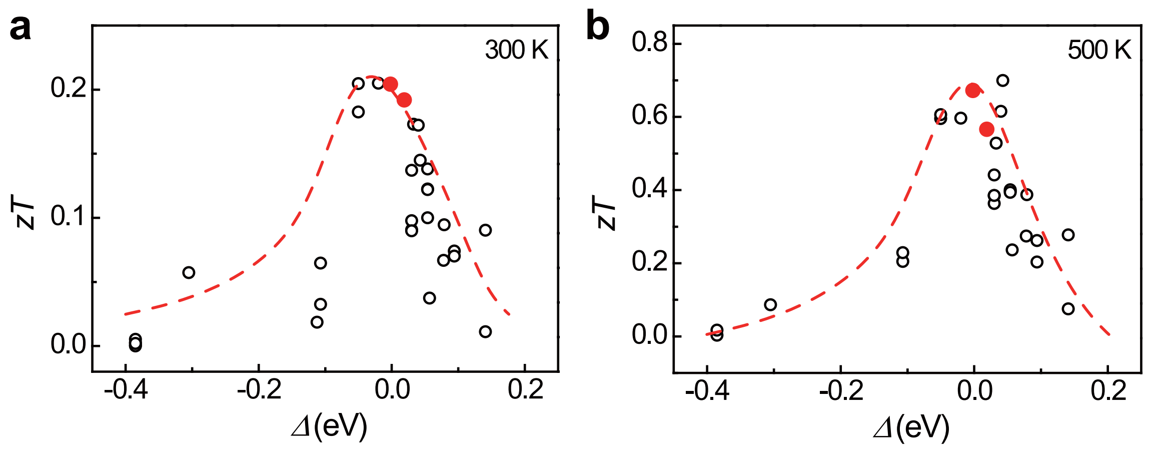

Supplementary Figure 6. Experimental zT values[1-18] of $CaAl_2Si_2$-type Zintl compounds at 300 K (a) and 500 K (b) as a function of Δ. Mixtures or solid solutions $YbCd_{1.6}Zn_{0.4}Sb_2$ and $EuZn_{1.8}Cd_{0.2}Sb_2$ with zT values[2,3] above unity at high temperature are marked in red. The curve is guide to the eye, showing the maximum values corresponding to optimal carrier concentrations.

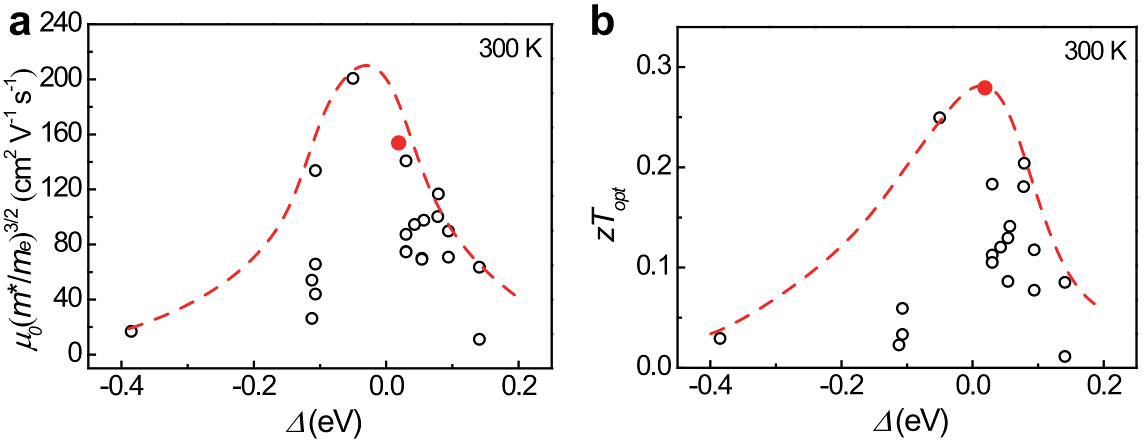

Supplementary Figure 7. Weighted mobility $\mu_0(m^*/m_e)^{3/2}$ (a) and optimum zT (b) at room temperature as a function of Δ in $CaAl_2Si_2$-type Zintl compounds. The $\mu_0(m^*/m_e)^{3/2}$ values and optimum zT values are simulated based on experimental data from references[2,4-7,9-15,18] using the single parabolic band model under acoustic phonon scattering mechanism. Compound $YbCd_{1.6}Zn_{0.4}Sb_2$ with zT value[2] above 1 at high temperature is marked in red. The curve is guide to the eye, showing the maximum values corresponding to optimal carrier concentrations. The insufficiency of the single parabolic band model in describing the anisotropic valence bands in $CaAl_2Si_2$ type Zintl compounds and the varying carrier concentrations from different references means that the data points for unoptimized carrier concentrations will fill up the whole area below the red curve.

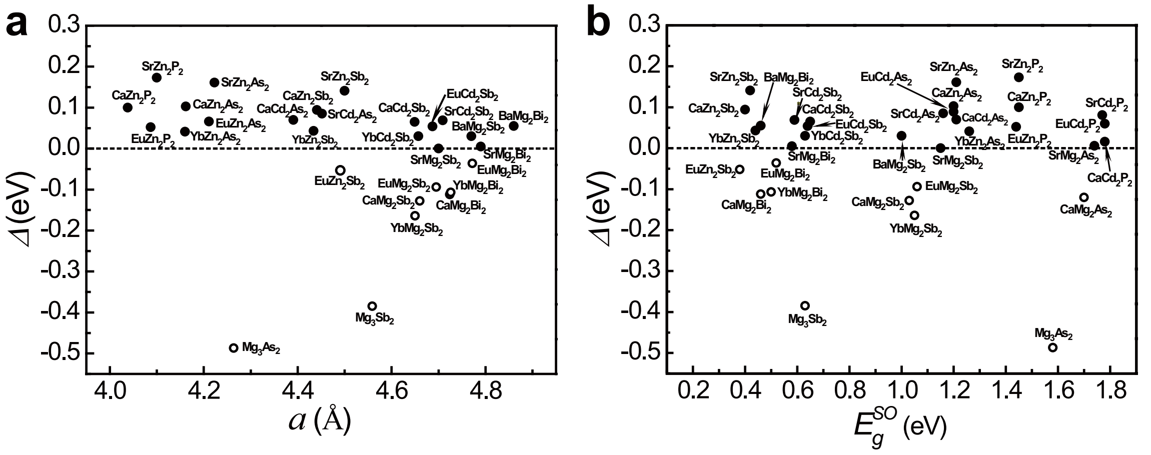

Supplementary Figure 8. Crystal field splitting energy *Δ* as a function of lattice constant *a* (a) and calculated energy gap including SOC E_g^{SO} (b) in $CaAl_2Si_2$-type Zintl compounds. The dotted line is the zero line. Filled circles are positive *Δ* compounds. Empty circles are negative *Δ* compounds. From the solid solution map, we find that there are still lots of potential TE candidates including $(Eu,Sr)Zn_2Sb_2$, $(Eu,Ca)Zn_2Sb_2$, $(Eu,Yb)Zn_2Sb_2$, $(Yb,Ba)Mg_2Sb_2$, $Ca(Mg,Cd)_2Sb_2$, $Yb(Mg,Zn)_2Sb_2$, $(Ca,Ba)Mg_2Bi_2$, etc.

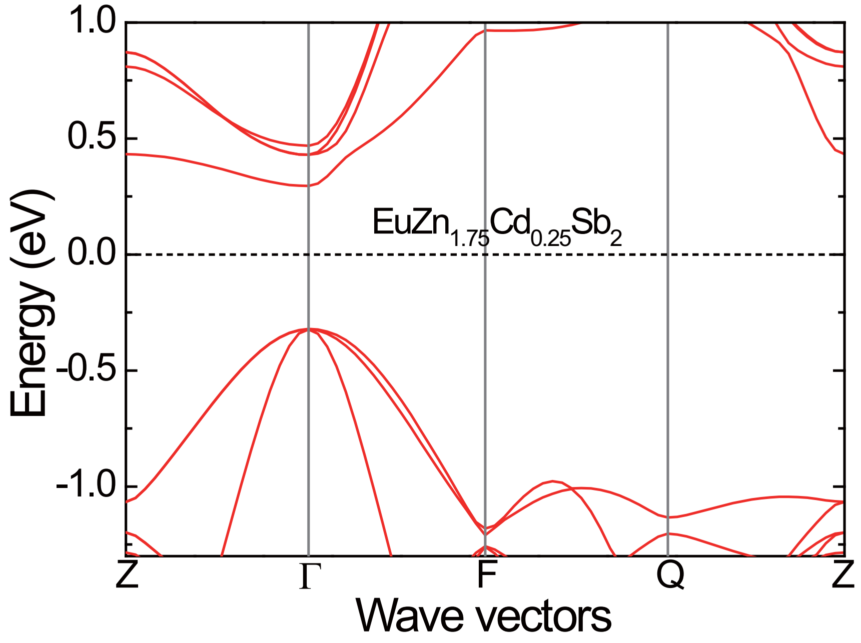

Supplementary Figure 9. Band structure of solid solution $EuZn_{1.75}Cd_{0.25}Sb_2$ with nearly zero Δ value.

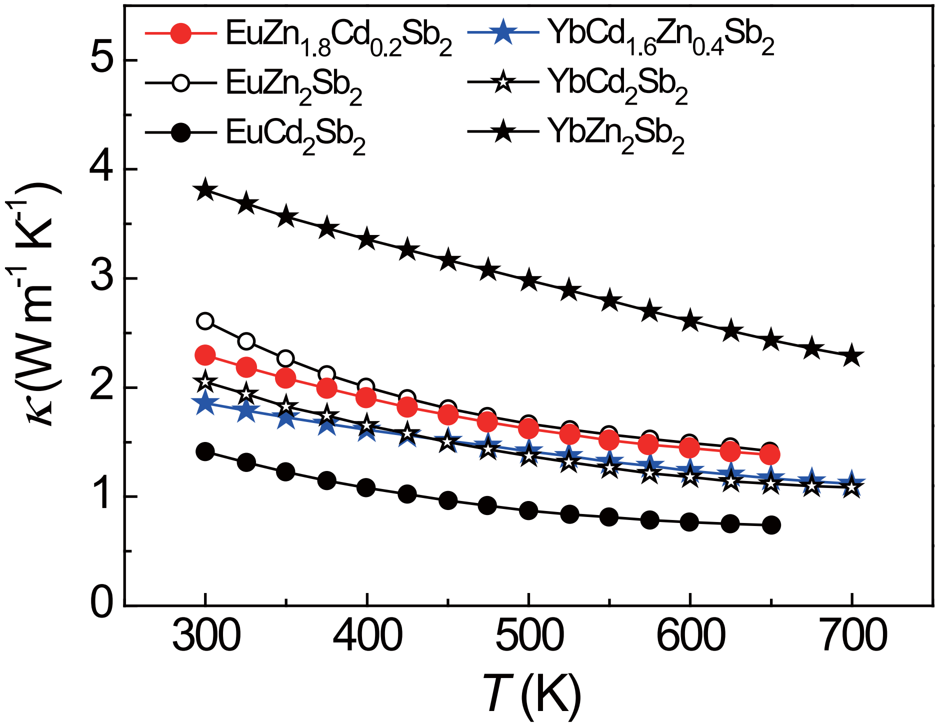

Supplementary Figure 10. Temperature dependence of thermal conductivity κ of $YbZn_2Sb_2$ ($EuZn_2Sb_2$), $YbCd_2Sb_2$ ($EuCd_2Sb_2$), and their solid solutions $YbCd_{1.6}Zn_{0.4}Sb_2$ and $EuZn_{1.8}Cd_{0.2}Sb_2$[2,3].

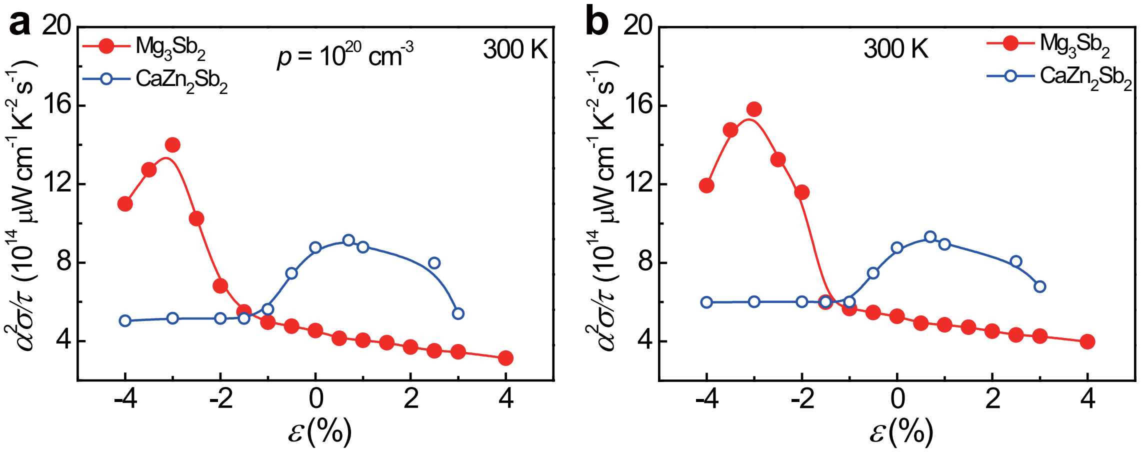

Supplementary Figure 11. Theoretical $\alpha^2\sigma/\tau$ at a given hole concentration p=1×10^{20}cm^{-3} (a) and at optimal hole concentration (b) at room temperature as a function of biaxial strain ε in two representative $CaAl_2Si_2$-type Zintl compounds, Mg_3Sb_2 and $CaZn_2Sb_2$. The solid lines represent fitted curves using a *B* spline.

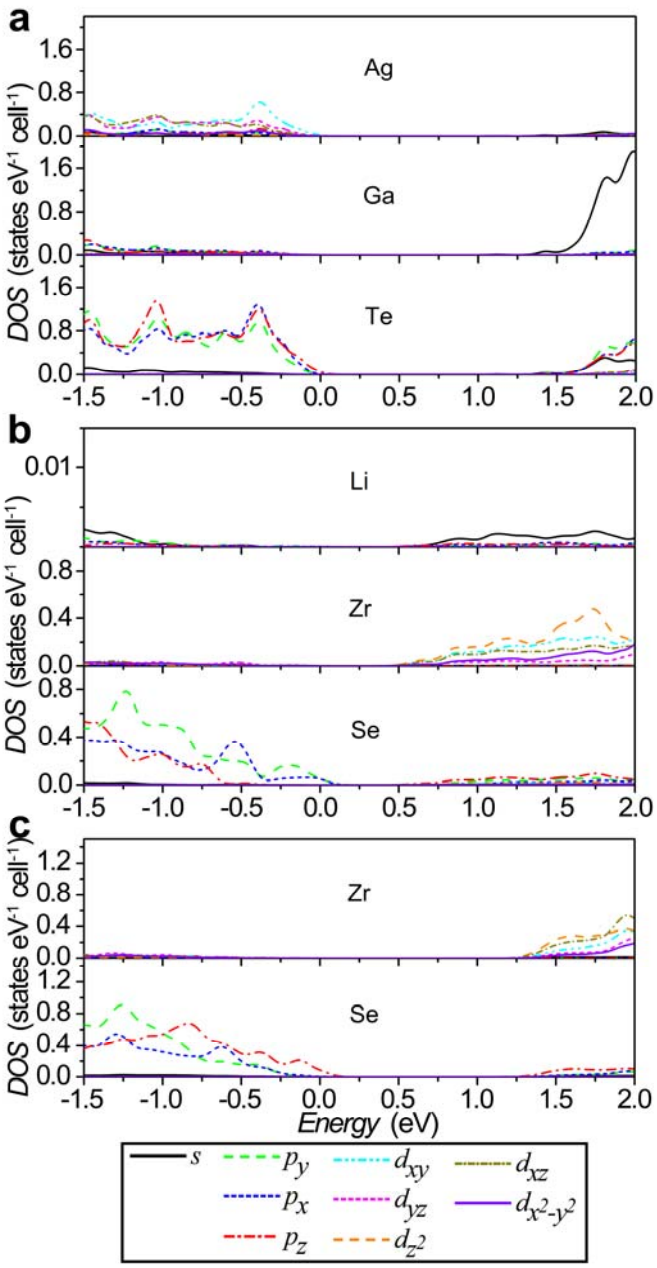

Supplementary Figure 12. Calculated orbital-projected density of states of (a) chalcopyrite compound $AgGaTe_2$, (b) lithium intercalated metal dichalcogenide $LiZrSe_2$, and (c) metal dichalcogenide ZrS_2.

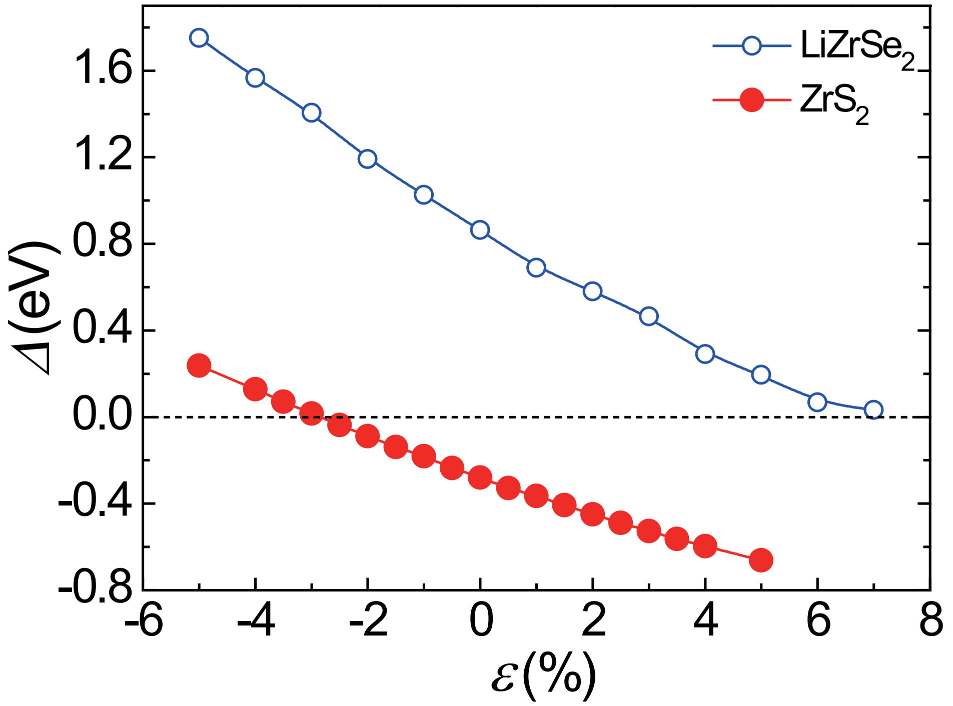

Supplementary Figure 13. Crystal field splitting energy *Δ* as a function of biaxial strain *ε* in representative metal dichalcogenide ZrS_2 and lithium intercalated metal dichalcogenide $LiZrSe_2$. The solid lines represent fitted curves using a *B* spline.

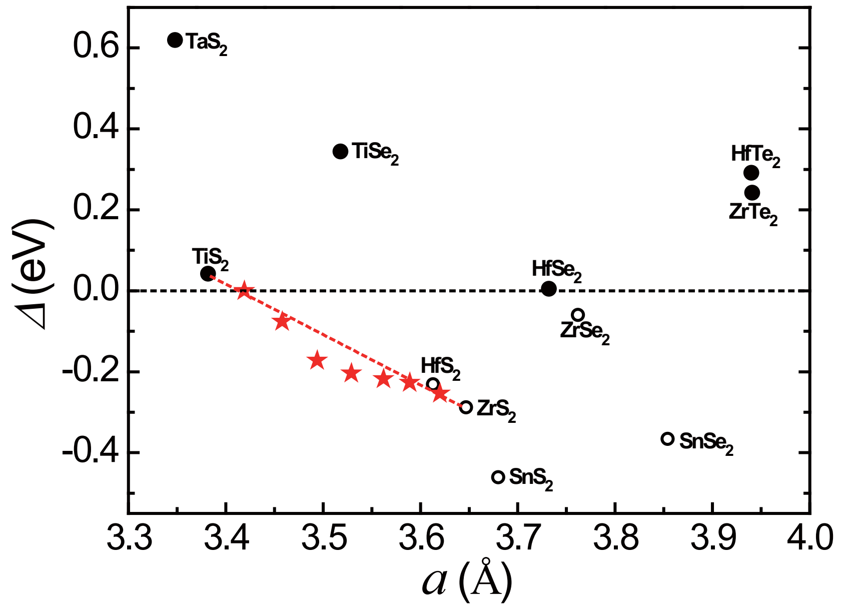

Supplementary Figure 14. Crystal field splitting energy Δ as a function of lattice constant a in carefully selected metal dichalcogenides. The red stars with trend line represent mixtures or solid solutions between two metal dichalcogenides with positive and negative Δ values. The dotted line is the zero line. Filled circles are positive Δ compounds. Empty circles are negative Δ compounds.

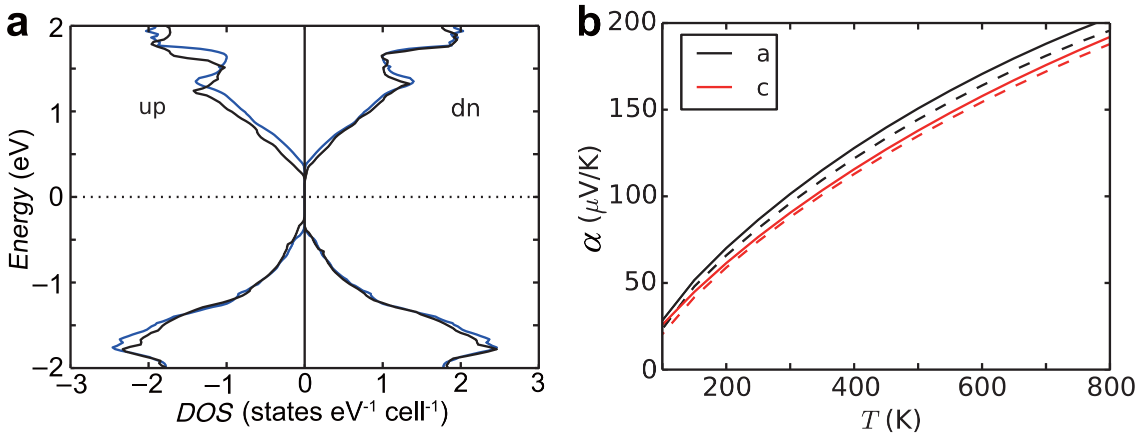

Supplementary Figure 15. Electronic properties of $EuZn_2Sb_2$. (a) Density of states around the Fermi level. The black lines are for spin-polarized all-electron calculations and the blue lines (which are identical for the two spin channels) are for non-spin-polarized PAW[19] calculations. (b) Calculated Seebeck coefficient at $p = 10^{20}$ cm^{-3} along the *a* and *c* directions. The full lines correspond to all-electron calculations. The Seebeck coefficients of spin-channels have been averaged as $\alpha = (\alpha\uparrow\sigma\uparrow + \alpha\downarrow\sigma\downarrow)/(\sigma\uparrow + \sigma\downarrow)$. The dashed lines correspond to non-spin-polarized PAW calculations.

Supplementary Tables

Supplementary Table 1. Room temperature thermoelectric transport properties of $CaAl_2Si_2$-type Zintl compounds[2,4-7,9-16,18]. Density of states effective mass m^*, intrinsic mobility μ_0, and weighted mobility $\mu_0(m^*/m_e)^{3/2}$ listed in this table are calculated from Hall data using single parabolic band model under acoustic phonon scattering mechanism. Hall data of Mg_3Bi_2 marked in red was not used for modeling weighted mobility and optimal zT since Mg_3Bi_2 is a semimetal indicated by band gap calculation with spin orbit coupling (see Supplementary Table 2).

Systems	α (μV K^{-1})	σ (10^4 S m^{-1})	p (10^{20} cm^{-3})	μ_H (cm^2 V^{-1} s^{-1})	m^*/m_e	$\mu_0(m^*/m_e)^{3/2}$ (cm^2 V^{-1} s^{-1})
$YbCd_2Sb_2$[2]	116.47	4.572	0.390	73	0.74	74.77
$YbCd_{1.6}Zn_{0.4}Sb_2$[2]	102.04	11.405	0.600	118	0.83	153.73
$YbCdZnSb_2$[2]	68.14	14.284	0.860	103	0.67	116.63
$YbCd_{0.8}Zn_{1.2}Sb_2$[2]	55.87	15.090	0.910	104	0.56	100.25
$YbCd_{0.4}Zn_{1.6}Sb_2$[2]	41.08	20.311	1.030	123	0.45	97.52
$YbCd_2Sb_2$[4]	122.02	4.889	0.430	72	0.84	87.30
Mg_3Sb_2[18]	591.00	0.004	0.002	11.25	1.21	16.89
$YbCd_2Sb_2$[5]	116.18	4.565	0.390	73	0.74	74.49
$EuCd_2Sb_2$[6]	216.00	1.151	0.044	182.4	0.45	69.88
$YbZn_2Sb_2$(332K)[7]	147.78	4.450	0.323	87.6	0.82	94.54
$CaZn_2Sb_2$[9]	120.00	5.000	0.330	99	0.69	89.77
$EuZn_2Sb_2$[10]	121.98	11.363	0.277	257	0.63	200.68
$CaZn_2Sb_2$[11]	120.00	4.219	0.310	83	0.66	70.70
$SrZn_2Sb_2$[13]	159.00	2.220	0.150	95	0.61	63.42
$YbCd_2Sb_2$[14]	168.16	3.333	0.087	326.7	0.46	140.77
$EuCd_2Sb_2$[14]	227.18	1.111	0.044	159.3	0.49	69.15
$SrZn_2Sb_2$[15]	167.47	0.356	0.050	45	0.32	11.09
$CaMg_2Bi_2$[12]	287.00	0.212	0.011	119	0.32	26.21
$YbMg_2Bi_2$[12]	240.00	0.614	0.029	131	0.42	44.00
$YbMg_2Bi_2$[12]	190.00	1.639	0.083	123	0.55	65.76
$CaMg_2Bi_2$[12]	115.00	3.279	0.200	105	0.47	54.11
$YbMg_2Bi_2$[12]	83.00	11.765	0.460	175	0.55	133.68
Mg_3Bi_2(324K)[16]	57.30	17.149	2.890	38.5		

Supplementary Table 2. Lattice parameters, calculated crystal field splitting energy *Δ*, absolute values of theoretical valence band splitting energy with spin orbit coupling (SOC) |*Δ*(SO)|, and calculated energy gaps E_g without SOC and E_g^{SO} with SOC of $CaAl_2Si_2$-type Zintl compounds. Potential TE compounds with nontoxic, low cost, and earth-abundant elements are marked in red color. *a* and *c* are experimental data from ICSD database[20], while the internal co-ordinates are determined by energy minimization. Lattice parameters and atomic positions of $EuZn_2Sb_2$, $EuCd_2Sb_2$, $YbZn_2Sb_2$, and $YbCd_2Sb_2$ are relaxed using HSE06 functional[21] with the aim to keep consistent with solid solution calculations.

$CaAl_2Si_2$-type Zintl compounds (Space group $P\bar{3}m1$)						
Compounds	*a* (Å)	*c* (Å)	*Δ* (eV)	|*Δ*(SO)|(eV)	E_g (eV)	E_g^{SO} (eV)
$CaZn_2P_2$	4.038	6.836	0.1		1.6	1.45
$CaCd_2P_2$	4.277	7.031	0.016		1.92	1.78
$SrZn_2P_2$	4.1	7.101	0.173		1.58	1.45
$SrCd_2P_2$	4.338	7.269	0.081		1.84	1.77
$EuZn_2P_2$	4.087	7.01	0.052		1.59	1.44
$EuCd_2P_2$	4.325	7.179	0.06		1.93	1.78
Mg_3As_2	4.264	6.738	-0.487	0.415	1.58	1.58
$CaMg_2As_2$	4.34	7.13	-0.12	0.09	1.82	1.7
$CaZn_2As_2$	4.162	7.01	0.103	0.108	1.36	1.2
$CaCd_2As_2$	4.391	7.184	0.07	0.073	1.29	1.21
$SrMg_2As_2$	4.41	7.41	0.006	0.054	1.91	1.74
$SrZn_2As_2$	4.223	7.268	0.161	0.128	1.36	1.21
$SrCd_2As_2$	4.452	7.416	0.085	0.082	1.24	1.16
$YbZn_2As_2$	4.16	6.961	0.041	0.085	1.46	1.26
$EuZn_2As_2$	4.211	7.181	0.066	0.093	1.37	1.21
$EuCd_2As_2$	4.45	7.35	0.089	0.09	1.29	1.2
Mg_3Sb_2	4.559	7.243	-0.385	0.299	0.64	0.63
$CaMg_2Sb_2$	4.66	7.58	-0.128	0.1	1.27	1.03
$CaZn_2Sb_2$	4.441	7.464	0.094	0.098	0.68	0.4
$CaCd_2Sb_2$	4.649	7.597	0.065	0.078	0.93	0.65
$SrMg_2Sb_2$	4.7	7.83	0	0.056	1.43	1.15
$SrZn_2Sb_2$	4.5	7.716	0.141	0.123	0.64	0.42
$SrCd_2Sb_2$	4.709	7.822	0.069	0.076	0.8	0.59
$BaMg_2Sb_2$	4.77	8.1	0.03	0.059	1.27	1
$EuMg_2Sb_2$	4.695	7.724	-0.094	0.085	1.31	1.06
$EuZn_2Sb_2$	4.49	7.5	-0.052	0.044	0.59	0.38
$EuCd_2Sb_2$	4.687	7.601	0.054	0.077	0.87	0.64
$YbMg_2Sb_2$	4.65	7.54	-0.164	0.126	1.3	1.05
$YbZn_2Sb_2$	4.434	7.41	0.043	0.036	0.7	0.44
$YbCd_2Sb_2$	4.657	7.546	0.03	0.055	0.9	0.63

Mg_3Bi_2	4.666	7.401	-0.305		0.31	0
$CaMg_2Bi_2$	4.724	7.651	-0.112	0.096	1.12	0.46
$SrMg_2Bi_2$	4.79	7.93	0.005	0.062	1.22	0.58
$BaMg_2Bi_2$	4.86	8.22	0.055	0.075	1.13	0.46
$EuMg_2Bi_2$	4.772	7.837	-0.036	0.064	1.22	0.52
$YbMg_2Bi_2$	4.726	7.645	-0.107	0.086	1.16	0.5

Supplementary Table 3. Calculated lattice parameters, crystal field splitting energy *Δ*, and calculated energy gap E_g without SOC of several MX_2 metal dichalcogenides with $P\bar{3}m1$ as space group. ZrS_2 and TiS_2 marked in red are two constituent compounds used for the validation of solid solution map (Supplementary Fig. 14). Lattice parameters and atomic positions of ZrS_2 and TiS_2 are relaxed using the same method as solid solution calculations.

MX_2 metal dichalcogenides (Space group $P\bar{3}m1$)				
Compounds	*a* (Å)	*c*(Å)	*Δ* (eV)	E_g (eV)
TaS_2	3.348	5.928	0.619	0
ZrS_2	3.647	5.797	-0.288	1.24
TiS_2	3.382	5.633	0.042	0.32
$ZrSe_2$	3.762	6.143	-0.06	0.61
SnS_2	3.68	5.89	-0.461	1.97
$TiSe_2$	3.518	5.997	0.344	0
$ZrTe_2$	3.941	6.65	0.242	0
$SnSe_2$	3.854	6.111	-0.366	0.95
HfS_2	3.613	5.842	-0.231	1.73
$HfSe_2$	3.732	6.173	0.005	1.04
$HfTe_2$	3.94	6.687	0.291	0

Supplementary Table 4. Effective mass along different lattice directions, conductivity effective mass and the calculated carrier scattering time τ for *p*-type Mg_3Sb_2. Effective mass is calculated from *ab initio* band structure.

p-type Mg_3Sb_2			
$m_{xx}^{*}\ (m_e)$	$m_{zz}^{*}\ (m_e)$	$m_I^{*}\ (m_e)$	$\tau\ (\times 10^{-14} s)$
1.15	0.084	0.221	0.376

Supplementary Notes

Supplementary Note 1. Orbital characteristics of valence bands affected by crystal field effect

Schematic diagram of electronic bands at valence band maximum in $CaAl_2Si_2$-type Zintl compounds is exemplified in **Supplementary Fig. 1**. Doubly degenerate band $\Gamma(p_{x,y})$ and nondegenerate band $\Gamma(p_z)$ are mainly from $p_{x,y}$ and p_z orbitals of anions (see **Supplementary Fig. 2**). The seperation of $\Gamma(p_{x,y})$ and $\Gamma(p_z)$ is caused by the crystal field splitting of p orbitals into $p_{x,y}$ and p_z orbitals. The crystal field splitting parameter Δ, is defined as the energy difference between $\Gamma(p_{x,y})$ and $\Gamma(p_z)$.

Experimental reported layered $CaAl_2Si_2$-type Zintl compounds, including $YbCd_2Sb_2$, $YbZn_2Sb_2$, $EuZn_2Sb_2$, $EuCd_2Sb_2$, $CaZn_2Sb_2$, $SrZn_2Sb_2$, $CaMg_2Bi_2$, $YbMg_2Bi_2$, Mg_3Bi_2, Mg_3Sb_2, $Eu(Zn_{1-x}Cd_x)_2Sb_2$, $Yb(Zn_{1-x}Cd_x)_2Sb_2$, and $Yb_{0.75}Eu_{0.25}Cd_2Sb_2$ (calculated $\Delta = 0.04$ eV) are studied and discussed in this work.

For Yb and Eu elements, the PAW[19] potentials, in which f-electrons are kept frozen in the core, are used for first principles calculations. To validate this approach we performed spin-polarized calculations on $EuZn_2Sb_2$ using WIEN2k[22]. The mBJ potential[23] was applied and a $U = 10$ eV was added for the Eu 4f-states as well as a $U = 4$ eV for the Zn 3d-states. **Supplementary Figure 15** illustrates how the localized nature of the Eu 4f-states leads to a very small magnetic splitting around the Fermi level. Consequently, the calculated transport coefficients with the two methods (PAW f-frozen core and all-electron spin polarized) are in very good internal agreement.

Supplementary Note 2. The effect of spin orbit coupling

As indicated in **Supplementary Fig. 1**, spin orbital coupling (SOC) will further split doubly degenerate $p_{x,y}$ energy level into two electronic states: one still lies at the valence band maximum; the other will shift downwards and might be far away from band edge depending on the degree of spin orbit coupling. Although SOC further lifts the degeneracy of $p_{x,y}$ band which will degrade the electrical transport properties, there still exists two electronic states at valence band maximum, each of which possesses the degeneracy of 2 (include spin), that can also be tuned by solid solution method and strain engineering. Since valence band maximum of $CaAl_2Si_2$-type Zintl compounds is mainly from anions, valence band splitting is mainly determined by spin orbit coupling of anion elements.

The energy difference between top electronic state split from $p_{x,y}$ band and the electronic state from p_z band is defined as Δ(SO). The spin orbit splitting energy of $p_{x,y}$ band is labeled as Δ_{SO}. The calculated absolute values of Δ(SO) ($|\Delta(SO)|$) of $CaAl_2Si_2$-type Zintl compounds are listed in **Supplementary Table 2**. We find that $|\Delta(SO)|$ and $|\Delta|$ values of most compounds containing relatively heavy elements Sb and Bi (with large $\Delta_{SO} > 0.6$ eV) discussed in this paper are comparable. It means that strong SOC will push one state far away from band edge

but will only have minor effects on the valence band splitting energy Δ(SO) at band edge. Moreover, for compounds with light elements SOC only has minor effects on the electronic bands at band edges. For this situation, it is reasonable to only consider crystal field effect for electronic structure calculations.

In order to check the effect of SOC on electronic transport properties, the electronic structure and transport calculation including SOC effect were carried out for Mg_3Sb_2 by combining WIEN2k[22] and BoltzTrap[24]. The zone samplings were done with uniform 36×36×24 *k* mesh grids, while a cut-off for the basis set RK_{max} = 9 was used. TB-mBJ functional[23] was adopted for electronic structure calculations. The dependence of calculated power factor including SOC at room temperature on valence band splitting energy Δ(SO) is shown in **Supplementary Fig. 4**. As expected, theoretical power factor with SOC decreases compared with that without SOC (see **Supplementary Fig. 5a**) due to lifting the degeneracy of $p_{x,y}$ band; however, it still can be tuned by valence splitting energy Δ(SO) and shows a peak while Δ(SO) approaches zero, fully consistent with the trend only including crystal field effect. The above result proves that SOC effect will not change the main idea of orbital engineering. Therefore, to be simplified it is rational to only include crystal field effect in this work.

Supplementary Note 3. Theoretical power factors and their dependence on crystal field splitting

The crystal field splitting energy Δ in **Fig. 2a** in main text was varied by slightly tuning lattice parameters *a* and *c*. Considering the relatively minor changes to the crystal structure for each step, the electron relaxation time τ was assumed to be independent of Δ in this work. For each Δ value, the theoretical power factor $\alpha^2\sigma/\tau$ at a given hole concentration $p = 10^{20}$ cm^{-3} and optimal hole concentration at different temperatures were calculated and plotted in **Supplementary Figs 3a and 5**.

Supplementary Note 4. Calculation details of solid solutions

Solid solution calculations of $CaAl_2Si_2$-type Zintl compounds are carried out in a supercell with 20 atoms (2×2×1 unit cell). Here we use HSE06 functional[21] for crystal structure optimization. The reason why we use HSE06 functional is that the electronic states at valence band maximum are sensitive to the structural parameters and HSE06 functional can give relatively reasonable structural parameters close to experimental values. Considering the computational cost, a Hellmann-Feynman force convergence criterion of 0.05 eV Å^{-1} was used. A 4×4×4 Monkhorst-Pack *k* mesh and a 9×9×9 Monkhorst-Pack *k* mesh were used for crystal structure optimization and electronic structure calculations, respectively. Take $Eu(Zn_{1-x}Cd_x)_2Sb_2$ as an example. There are 8 equivalent positions for Zn/Cd atoms, thus the possible atomic concentrations of Cd are multiples of 1/8. The crystal structure of the supercell with the lowest energy is used for further calculations and analysis. The calculation method of electronic structure is the same as mentioned in the main text.

Solid solution calculations of layered metal dichalcogenides are carried out in a supercell with 24 atoms (2×2×2 unit cell). A 6×6×4 Monkhorst-Pack *k* mesh was used for crystal structure

optimization. Crystal structures are relaxed using PBE functional[25]. In layered metal dichalcogenides, interlayer *van der waals* interaction is very important. Thus, *Van der Waals* interaction was taken into account during structural relaxation at the vdW-DF level with optB86b[26] for exchange functional (optB86b-vdW). Lattice parameters and atomic positions were optimized until the maximum Hellmann-Feynman force acting on each atom is less than 0.005 eV $Å^{-1}$. The crystal structure of the supercell with the lowest energy is used for further calculations and analysis. The calculation method of electronic structure is the same as mentioned in the main text.

Supplementary Note 5. Calculation details of chalcopyrite $AgGaTe_2$, metal dichalcogenides, and lithium intercalated metal dichalcogenide $LiZrSe_2$

The crystal structures of $AgGaTe_2$ and $LiZrSe_2$ were relaxed using HSE06 functional[5]. An energy convergence criterion of 10^{-4} eV and a Hellmann-Feynman force convergence criterion of 0.008 eV $Å^{-1}$ were adopted for the calculations of $AgGaTe_2$ and $LiZrSe_2$. For $AgGaTe_2$, A 4×4×4 Monkhorst-Pack *k* mesh and a 10×10×10 Monkhorst-Pack *k* mesh were used for crystal structure optimization and electronic structure calculations, respectively. Electronic structure calculation of $AgGaTe_2$ was implemented by combining the mBJ potential and the well-established PBE+*U* method[23,27], and U = 2 eV was applied on Ag 4*d* states. The electronic structure calculation method of $LiZrSe_2$ is the same as that of $CaAl_2Si_2$-type zintl compounds stated in the main text.

The crystal structures of layered metal dichalcogenides were relaxed using PBE functional[25]. *Van der Waals* interaction was taken into account during structural relaxation at the vdW-DF level with optB86b[26] for exchange functional (optB86b-vdW). The electronic structure calculation method of metal dichalcogenides is the same as that of $CaAl_2Si_2$-type zintl compounds.

Supplementary Note 6. Calculation details of carrier scattering time for zT under biaxial strain effect

Due to the anisotropic band at valence band maximum, the carrier's scattering time τ is estimated from the relation $\mu = e\tau / m_I^*$, where μ is the carrier mobility, m_I^* is inertial or conductivity effective mass, and e is the elementary charge. In trigonal *p*-type Mg_3Sb_2, m_I^* can be expressed as $m_I^* = 3/(2/m_{xx}^* + 1/m_{zz}^*)$, where m_{xx}^* and m_{zz}^* are effective mass along x and z directions, respectively. Effective mass tensor m_{ij}^* is defined as $m_{ij}^* = \hbar^2[\partial^2 E(k)/\partial k_i \partial k_j]^{-1}$. m_{xx}^* and m_{zz}^* of *p*-type Mg_3Sb_2 are evaluated from *ab initio* band structure and thereby m_I^* is calculated using the above formula (see **Supplementary Table 4**). Using experimental reported mobility[1] μ = 30 $cm^2\,V^{-1}\,s^{-1}$ and calculated m_I^*, we can

obtain the constant carrier scattering time τ = 3.76×10^{-15} s for *p*-type Mg_3Sb_2. Under the framework of the constant carrier scattering time approximation, the carrier scattering time τ is assumed to be a constant, independent of the carrier concentration and temperature.

Supplementary References

1. Bhardwaj, A. *et al.* Mg_3Sb_2-based Zintl compound: a non-toxic, inexpensive and abundant thermoelectric material for power generation. *RSC Adv.* **3,** 8504-8516 (2013).
2. Wang, X.-J. *et al.* Synthesis and high thermoelectric efficiency of Zintl phase $YbCd_{2-x}Zn_xSb_2$. *Appl. Phys. Lett.* **94,** 092106 (2009).
3. Zhang, H. *et al.* Thermoelectric properties of $Eu(Zn_{1-x}Cd_x)_2Sb_2$. *Dalton Trans.* **39,** 1101-1104 (2010).
4. Guo, K. *et al.* Enhanced thermoelectric figure of merit of Zintl phase $YbCd_{2-x}Mn_xSb_2$ by chemical substitution. *Eur. J. Inorg. Chem.* **2011,** 4043-4048 (2011).
5. Cao, Q.-G. *et al.* Zintl phase $Yb_{1-x}Ca_xCd_2Sb_2$ with tunable thermoelectric properties induced by cation substitution. *J. Appl. Phys.* **107,** 053714 (2010).
6. Zhang, H. *et al.* Synthesis and properties of $CaCd_2Sb_2$ and $EuCd_2Sb_2$. *Intermetallics* **18,** 193-198 (2010).
7. Zevalkink, A. *et al.* Nonstoichiometry in the Zintl phase $Yb_{1-\delta}Zn_2Sb_2$ as a route to thermoelectric optimization. *Chem. Mater.* **26,** 5710-5717 (2014).
8. Condron, C. L., Kauzlarich, S. M., Gascoin, F. & Snyder, G. J. Thermoelectric properties and microstructure of Mg_3Sb_2. *J. Solid State Chem.* **179,** 2252-2257 (2006).
9. Stark, D. & Snyder, G. J. The synthesis of $CaZn_2Sb_2$ and its thermoelectric properties. *in Proc. 21th Inter. Conf., Thermoelectrics* 181-184 (2002).
10. Zhang, H. *et al.* A new type of thermoelectric material, $EuZn_2Sb_2$. *J. Chem. Phys.* **129,** 164713 (2008).
11. Gascoin, F., Ottensmann, S., Stark, D., Haïle, S. M. & Snyder, G. J. Zintl Phases as thermoelectric materials: tuned transport properties of the compounds $Ca_xYb_{1-x}Zn_2Sb_2$. *Adv. Funct. Mater.* **15,** 1860-1864 (2005).
12. May, A. F. *et al.* Thermoelectric transport properties of $CaMg_2Bi_2$, $EuMg_2Bi_2$, and $YbMg_2Bi_2$. *Phys. Rev. B* **85,** 035202 (2012).
13. Toberer, E. S., May, A. F., Melot, B. C., Flage-Larsen, E. & Snyder, G. J. Electronic structure and transport in thermoelectric compounds AZn_2Sb_2 (A = Sr, Ca, Yb, Eu). *Dalton Trans.* **39,** 1046-1054 (2010).
14. Zhang, H. *et al.* Thermoelectric properties of $Yb_xEu_{1-x}Cd_2Sb_2$. *J. Chem. Phys.* **133,** 194701 (2010).
15. Zhang, H. *et al.* Thermoelectric properties of polycrystalline $SrZn_2Sb_2$ prepared by spark plasma sintering. *J.Electron.Mater.* **39,** 1772-1776 (2010).
16. Ponnambalam, V. & Morelli, D. On the thermoelectric properties of Zintl compounds $Mg_3Bi_{2-x}Pn_x$ (Pn = P and Sb). *J.Electron.Mater.* **42,** 1307-1312 (2013).
17. Ahmadpour, F., Kolodiazhnyi, T. & Mozharivskyj, Y. Structural and physical properties of

$Mg_{3-x}Zn_xSb_2$ (x=0–1.34). *J. Solid State Chem.* **180,** 2420-2428 (2007).

18. Kim, S. *et al.* Thermoelectric properties of Mn-doped Mg-Sb single crystals. *J. Mater. Chem. A* **2,** 12311-12316 (2014).
19. Blöchl, P. E. Projector augmented-wave method. *Phys. Rev. B* **50,** 17953-17979 (1994).
20. *Inorganic Crystal Structure Database (ICSD). http://icsd.fiz-karlsruhe.de/.*
21. Paier, J. *et al.* Screened hybrid density functionals applied to solids. *J. Chem. Phys.* **124,** 154709 (2006).
22. Blaha, P., Schwarz, K., Madsen, G., Kvasnicka, D., and Luitz, J. WIEN2k, *An Augmented Plane Wave + Local Orbitals Program for Calculating Crystal Properties* (Tech. Univ. Wien, Austria, 2001).
23. Tran, F. & Blaha, P. Accurate band gaps of semiconductors and insulators with a semilocal exchange-correlation potential. *Phys. Rev. Lett.* **102,** 226401 (2009).
24. Madsen, G. K. H. & Singh, D. J. BoltzTraP. A code for calculating band-structure dependent quantities. *Comput. Phys. Commun.* **175,** 67-71 (2006).
25. Perdew, J. P., Burke, K. & Ernzerhof, M. Generalized gradient approximation made simple. *Phys. Rev. Lett.* **77,** 3865-3868 (1996).
26. Klimeš, J., Bowler, D. R. & Michaelides, A. Van der Waals density functionals applied to solids. *Phys. Rev. B* **83,** 195131 (2011).
27. Zhang, Y. *et al.* Near-edge band structures and band gaps of Cu-based semiconductors predicted by the modified Becke-Johnson potential plus an on-site Coulomb U. *J. Chem. Phys.* **139,** 184706 (2013).
